# Cancer therapy: Attempt cure or manage drug resistance?

**DOI:** 10.1111/eva.12994

**Published:** 2020-08-06

**Authors:** Elsa Hansen, Andrew F. Read

**Affiliations:** ^1^ Department of Biology Center for Infectious Disease Dynamics Pennsylvania State University University Park PA USA; ^2^ Department of Entomology Pennsylvania State University University Park PA USA; ^3^ Huck Institutes of the Life Sciences Pennsylvania State University University Park PA USA

**Keywords:** cancer, containment, cure, drug resistance

## Abstract

Cancer treatment is often aimed at achieving rapid, large, and sustained reductions in tumor burden. Even when these strong responses are achieved, treatment frequently fails due to the emergence of drug‐resistant cell lineages. Over the last decade, a variety of authors have suggested that treatment should instead be aimed at containing resistance rather than curing the patient. That new philosophy poses a dilemma: how to choose between treatment regimens that can sometimes cure the patient and regimens that can delay progression but not cure the patient? Here, we investigate that choice. We define aspects of the evolution and ecology of tumor dynamics that determine whether it is better to attempt cure or to manage resistance. Even when it is possible to manage resistance and delay progression, this may not be the best treatment option. We show that the best option depends on how “cure” and “delaying progression” are prioritized, and how those priorities will vary among patients. We also discuss the difficulties of comparing in clinical trials traditional strategies that can sometimes successfully cure to alternative approaches where cure is not possible. More generally, where resistance management is possible, there are new challenges in communicating options to patients, setting treatment guidelines, and evaluating data from clinical trials.

## 1 INTRODUCTION

The aim of cancer therapy is to extend the patient's quality life span (Balis, 1998). Many drug treatment regimens are designed to achieve large, rapid reductions in tumor burden with a view to eliminating all cancer cells (Lonial & Anderson, 2014; Waks & Winer, 2019). Sometimes this approach is successful, and cure is achieved. But frequently, even when treatment initially shrinks the tumor, this approach fails because the tumor grows back and is no longer responsive to the original drug regimen (Gottesman, 2002; Nikolaou, Pavlopoulou, Georgakilas, & Kyrodimos, 2018). A long‐held belief is that, even if the tumor recurs, maximizing the initial tumor response is the best approach because it will—at the very least—delay progression (Barlogie et al., 2008; Burzykowski et al., 2008; Goring et al., 2017; van de Velde et al., 2007). If so, regardless of the outcome, treating to rapidly reduce tumor burdens increases the patient's quality life span (Gill & Sargent, 2006).

However, dramatically shrinking the tumor may not be the best way to delay progression (Das Thakur et al., 2013; Studer et al., 2014; Teply et al., 2018). Recent theory from evolution and ecology suggests that there may be better ways to control populations of cancer cells (Gatenby, Silva, Gillies, & Frieden, 2009; Hansen, Woods, & Read, 2017; Read, Day, & Huijben, 2011; Wale et al., 2017). When the emergence of drug‐resistant cell lineages makes it impossible to control tumor burden, then slowing the expansion of the drug‐resistant population should delay progression. This thinking shifts the goal of treatment from “cure” to “resistance management.”

Here, we focus on “resistance management” strategies that use competition to suppress the expansion of the drug‐resistant population (Enriquez‐Navas et al., 2016; Hansen et al., 2017). The idea behind competitive suppression is that different cell populations compete with each other for resources required to multiply and survive (Garber, 2015; Hsu & Sabatini, 2008). If competitive suppression is effective, then the resistant cell population will grow more slowly in the presence of sensitive cells, and tumor control will be maintained for longer. Instead of maximizing tumor shrinkage, the proposition is to deliberately maintain larger tumor burdens to ensure that there are sensitive cells to suppress the resistant population (Gatenby et al., 2009).

A recent example of this approach is “adaptive therapy” used to treat metastatic castrate‐resistant prostate cancer (Zhang, Cunningham, Brown, & Gatenby, 2017). This approach uses the same drug as standard therapy but applies it differently. Where standard therapy would continue treatment until maximal tumor response was achieved (and perhaps even longer), “adaptive therapy” only administers drug long enough to achieve a 50% reduction in tumor burden. At this point, further treatment is withheld until the tumor burden recovers to its initial (pretreatment) baseline, and then, the treatment cycle is repeated. This approach has shown promising results in a pilot clinical trial, with adaptive therapy greatly increasing time to progression over standard therapy (Zhang, Fishman, Brown, & Gatenby, 2019). This is an example of a resistant management strategy designed to use competition to delay progression. In essence, the drug is used to contain the sensitive tumor cells and the sensitive cells are used to contain the resistant tumor cells.

Successful resistance management treatment strategies often involve chronic control of cancer; cure is rarely—if ever—achieved. Cure, if it can be achieved, is normally the more preferable outcome (depending on side effects), but treating to “cure” may carry some risk. If treating to “cure” fails, then the cancer may progress more rapidly than if a resistance management strategy had been used because resistant cell lineages can expand unconstrained by competition with sensitive cells. This raises a dilemma. If there is a possibility of resistance developing to a drug (or combination of drugs), what should the aim of treatment be? Large reductions in tumor burden with the ultimate aim of cure? Or minimal reductions in tumor burden aimed at containing resistance for as long as possible?

Here we consider how to choose between “attempting cure” and “managing resistance.” Often, the choice requires prioritizing “cure” and “delaying progression.” The possibility of cure—even when resistance is present—is what makes this decision so difficult and what distinguishes the analysis we present here from the one presented earlier (Hansen et al., 2017). We argue that the decision‐making process reduces to two stages. The first stage is quantifying (a) the probability of cure and (b) the effect of resistance management on progression. The second stage is determining how “cure” and “delayed progression” are valued. There is no single way to do this, but how these outcomes are valued strongly impacts the treatment decision. Not being explicit about this valuation process can have negative consequences on the design and interpretation of clinical trials.

## 2 CONCEPTUAL FRAMEWORK

**FIGURE 1:**
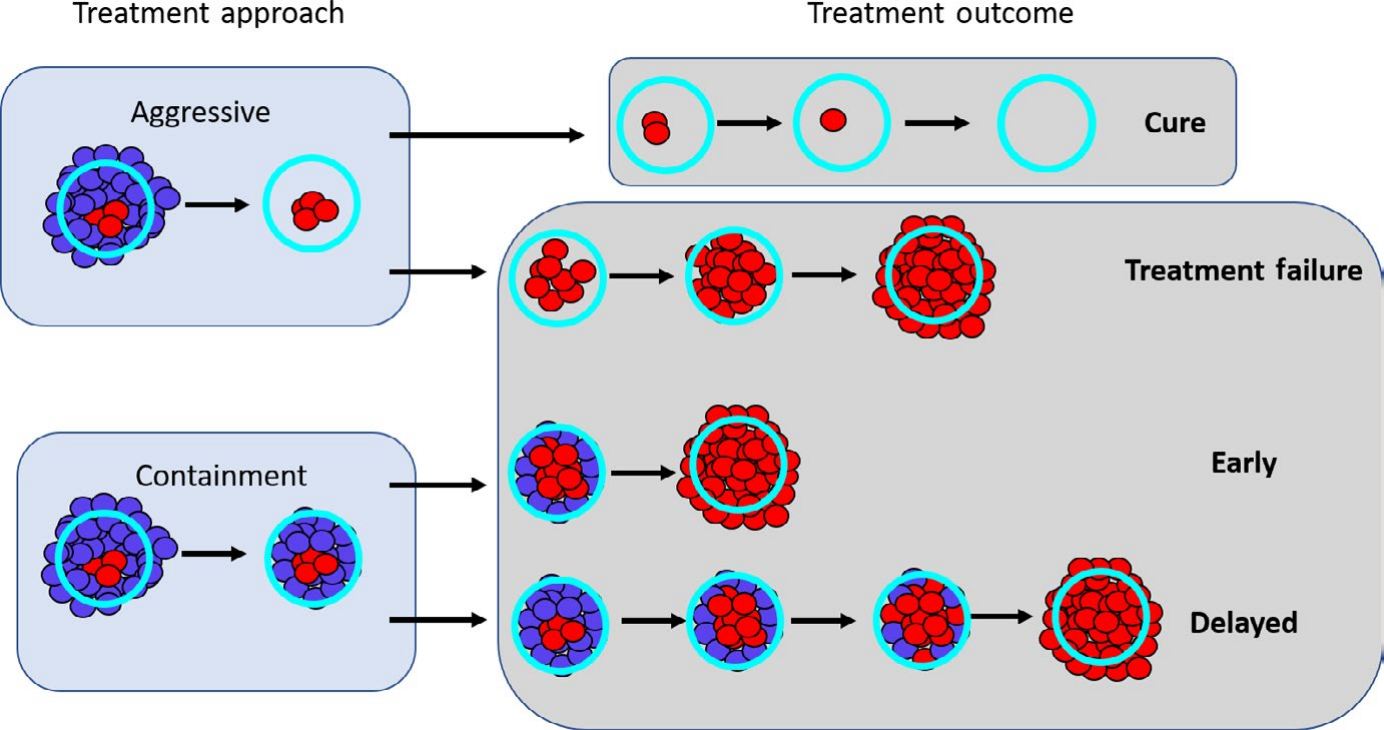
Conceptual framework. Once aggressive treatment immediately removes all drug‐sensitive cells, there are two possible outcomes. The first is that any remaining drug‐resistant population will eventually die out due to random effects. In this case, aggressive treatment cures the patient. The second possible outcome is that the remaining resistant population continues to grow and eventually exceeds the patient's baseline burden. When this occurs, the cancer has progressed. If containment is used, then the tumor is maintained at the patient's baseline burden for as long as possible. In this case, the resistant population will continue to grow until the tumor is predominantly resistant and grows to exceed the baseline. At this point, the cancer has progressed. Compared to aggressive treatment, containment may either lead to early or delayed progression

We begin by describing the conceptual framework we will use to compare “attempting cure” and “managing resistance” (Figure 1). This framework is a deliberately simplified caricature of tumor growth and is intended to focus on the fundamental issues. It is similar to the conceptual framework presented previously (Hansen et al., 2017) with the important distinction that it allows for the possibility of cure (see Box 1 for a description of the mathematical model). We assume that a tumor consists of drug‐sensitive and drug‐resistant cells. The size of a tumor at the beginning of treatment is the *baseline burden* *B*_*base*_. If it is necessary for immediate health reasons to first reduce a patient's tumor to an acceptable burden, then we will call this newly achieved acceptable burden the baseline burden. We further assume that the side effects of drugs are negligible and that the tumor only presents a risk to the patient if it progresses (i.e., grows beyond its baseline burden).

BOX 1 **Mathematical model of tumor dynamics and treatment**

To model tumor dynamics, we use a simple birth–death process (Ravindran, Phillips, & Solberg, 1987). This allows us to account for stochastic effects that can eventually lead to cure. We will assume that the tumor consists of two different cell populations: a drug‐resistant population *R*, which is completely resistant to drug treatment, and a drug‐sensitive population *S*. The rate of cell replication (i.e., the birth rate) will depend on whether aggressive treatment or containment is being used.

Within our conceptual framework, aggressive treatment immediately removes all drug‐sensitive cells and so the total tumor is instantly reduced to just the drug‐resistant population. We model the birth rate under aggressive treatment as

$$B_{A}\left(R\right)=r\left(1-\delta R\right)R,$$

where *r* is the probability of replication in the absence of competition, and *δ* is the competition coefficient. The amount of competition depends on the size of the resistant population and decreases the replication rate by a factor (1 − *δR*).

Under containment, the total tumor burden is maintained at the baseline burden *B*_*base*_ and consists of both resistant and sensitive cells. In this case, the birth rate for the resistant population takes a slightly more complicated form for two reasons. First, resistant cells will experience competition from sensitive cells and other resistant cells. Since the entire tumor population *B*_*base*_ is contributing to competition, replication of the resistant population will be reduced by a factor (1 − *δB*_*base*_) instead of (1 − *δR*). Second, we allow for the possibility that the sensitive cells could generate resistant mutants. If the sensitive replication rate is *r*(1 − *δB*_*base*_)*S* and the probability of mutating to resistance is *ɛ*, then the rate of mutational input from the sensitive population to the resistant population is *ɛr*(1 − *δB*_*base*_)*S*. Since the total tumor size is *B*_*base*_ during containment, the amount of mutational input can also be rewritten as *ɛr*(1 − *δB*_*base*_)(*B*_*base*_ − *R*). Therefore, under containment the birth rate of the resistant population is

$$B_{C}\left(R\right)=r\left(1-\delta B_{\mathrm{base}}\right)R+\varepsilon r\left(1-\delta B_{\mathrm{base}}\right)\left(B_{\mathrm{base}}-R\right).$$

We assume that there is a constant probability *µ* of cell death and that this is the same for both treatment strategies. In other words, the death rate is *D*(*R*) = *µR*, for both aggressive treatment and containment. These specific birth and death rates can be substituted into the expressions in Box 3 to determine the probability of cure and the expected times to progression.

In an ideal situation, treating to cure leads to the immediate removal of all drug‐sensitive cells. We will call this *aggressive treatment*. If there are no resistant cells, then aggressive treatment results in immediate cure. If there is a small resistant population, then there is still some possibility that this population will die out due to random effects and the patient will be cured. It is also possible that the resistant population will grow and eventually exceed the patient's baseline burden. At this point, we say that the cancer has *progressed*. In this case, cure was not achieved even though cure was the aim.

The second approach, treating to manage resistance, uses competition to try to delay progression and contain the cancer for as long as possible. To maximize competition, we maintain the largest possible sensitive population—without allowing for progression. This means treating just enough to keep the tumor at its baseline burden. Eventually, treatment will no longer be able to contain the tumor because it will consist predominantly of resistant cells. At this point, progression occurs. We will call this method of leveraging competition to control the tumor, *containment*.

When aggressive treatment fails to cure the patient, the resulting time to progression can be compared to the time to progression under containment. We will compare time to progression between the two strategies by looking at the ratio of these times. Specifically,

(1) $$\left(\begin{array}{c} \text{Fold}\;\;\text{Change}\;\text{In}\\ \text{Progression}\;\text{Time} \end{array}\right)=\frac{\text{Expected}\;\text{Progression}\;\text{Time}\;\text{With}\;\text{Containment}}{\text{Expected}\;\text{Progression}\;\text{Time}\;\text{With}\;\text{Aggressive}\;\text{Treatment}}.$$

Due to random effects during cell replication and death, there will be a distribution of possible progression times for both aggressive treatment and containment. An important feature of the fold change in progression time is that it is a comparison of *expected* times to progression (i.e., the expected values of these distributions). In what follows, we use the expected progression times as a summary of these distributions. If another feature of these distributions is considered more important, then it could be used instead.

There are two possible outcomes. The first is that containment delays progression relative to aggressive treatment (fold change in progression time is >1). In this case, the resistance management strategy is successful. The second possible outcome is that containment actually leads to early progression (fold change in progression time is <1). Whether containment leads to early or delayed progression is heavily influenced by the evolution and ecology governing tumor dynamics and is discussed in the next section.

## 3 A PRIMER ON CONTAINMENT

Containment is designed to use drug‐sensitive cells to competitively suppress the expansion of the drug‐resistant population. The problem is that competition is not the only way that sensitive cells influence the resistant population. For example, when sensitive cells divide, mutations can occur that confer resistance. This means that containment will delay progression only if the overall effect of competitive suppression exceeds the overall effect of mutational input. If there is essentially no mutation, then containment will delay progression at least as long as aggressive treatment (how much longer will depend on how strong competition is). On the other hand, if competition is negligible and the probability of mutation is high, then progression will actually occur sooner with containment.

Theory suggests that the balance between competitive suppression and mutational input changes as the resistant population grows (Hansen et al., 2017). When the resistant population is small, containment actually accelerates the expansion of the resistant population because of mutational input from the sensitive population. Competitive suppression becomes the dominant effect only once the resistant population is large enough. The size of the resistant population where this switch occurs is called the balance threshold *R*_*balance*_ (see Box 2 for a derivation of *R*_*balance*_). This means that during containment, the resistant population can go through periods of time where its expansion is enhanced by the presence of sensitive cells and periods where its expansion is slowed. Whether or not containment delays progression depends on the net effect of these periods and is strongly influenced by the balance between mutation and competition. In particular, if the baseline burden is less than the balance threshold (*B*_*base*_ < *R*_*balance*_), then containment will never delay progression (Hansen et al., 2017).

BOX 2 **The balance threshold**

An important feature of our illustrative model is that the birth rate is minimized by containment whenever *R* is large and by aggressive treatment whenever *R* is low. Specifically,

$$\begin{array}{cc} B_{C}\left(R\right) & =r\left(1-\delta B_{\mathrm{base}}\right)R+\varepsilon r\left(1-\delta B_{\mathrm{base}}\right)\left(B_{\mathrm{base}}-R\right),\\ & =r\left(1-\delta R\right)R-r\delta \left(B_{\mathrm{base}}-R\right)R+\varepsilon r\left(1-\delta B_{\mathrm{base}}\right)\left(B_{\mathrm{base}}-R\right),\\ & =B_{A}\left(R\right)+\left[\varepsilon r\left(1-\delta B_{\mathrm{base}}\right)-r\delta R\right]\left(B_{\mathrm{base}}-R\right). \end{array}$$

Therefore, if we define the “balance threshold” (Hansen et al., 2017) by $R_{\mathrm{balance}}=\frac{\varepsilon }{\delta }\left(1-\delta B_{\mathrm{base}}\right)$then

$$\begin{array}{cc} B_{C}\left(R\right)<B_{A}\left(R\right) & if\;R>R_{\mathrm{balance}},\;\text{and}\\ B_{C}\left(R\right)>B_{A}\left(R\right) & if\;R<R_{\mathrm{balance}} \end{array}.$$

Another important feature of the balance threshold is that it is a function of the baseline burden *B*_*base*_. From the expression for *R*_*balance*_, it is clear that increasing the baseline burden will decrease the balance threshold.

## 4 THE CURE–PROGRESSION PLANE

**FIGURE 2:**
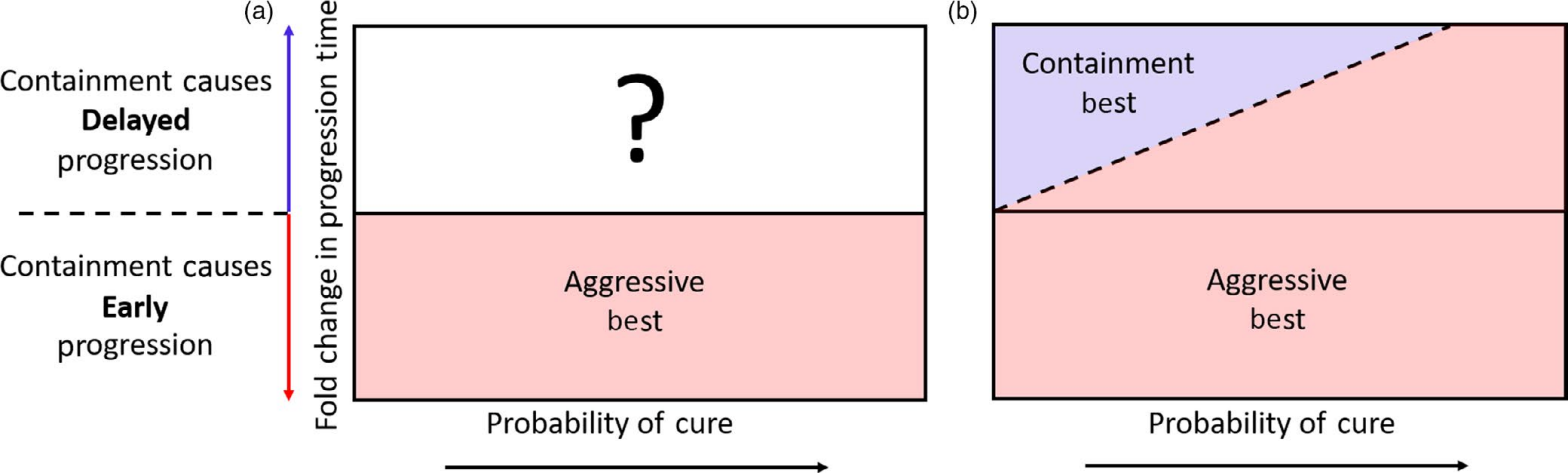
The cure–progression plane. Both the probability of cure (horizontal axis) and the effect of containment on progression time (vertical axis, defined in Equation 1) should be considered when making treatment decisions. Panel a: Our conceptual framework divides all possibilities into two sets: (i) Situations where containment leads to early progression. In this case, patients are located in the lower portion of the cure–progression plane (red shaded area) and aggressive treatment is best. (ii) Situations where containment delays progression. In this case, patients are located in the upper portion of the cure–progression plane (unshaded area) and it is unclear whether containment or aggressive treatment should be used. Panel b: If the value of cure and delayed progression is determined by how they change a patient's expected life span, then the decision boundary is an increasing line (dashed black line separating blue and red shaded areas). In this case, containment should be used whenever it results in a longer expected life span (blue shaded area). Solid black line is where the fold change in time to progression (Equation 1) is one

This framework naturally defines a cure–progression plane and allows us to partition all possible scenarios into two sets: one where aggressive treatment is obviously the preferred option and one where it is unclear whether containment or aggressive treatment should be used (Figure 2a). In the first set, containment leads to early progression and so aggressive treatment is best because it both maintains the possibility of cure and maximally delays progression. In this case, the patient lies in the lower half of the cure–progression plane (fold change in progression time is <1). In the second set, the decision is unclear and we are forced between attempting cure and delaying progression.

In practice, determining a patient's position on the cure–progression plane is very difficult. Complications arise for numerous reasons including, but not limited to, difficulties in measuring and detecting tumor burden, and inadequate models of tumor growth. Here, we ignore these difficulties (again, so we can concentrate on the fundamental issues) and focus on how to make treatment decisions under the assumption that we perfectly understand the tumor dynamics.

**FIGURE 3:**
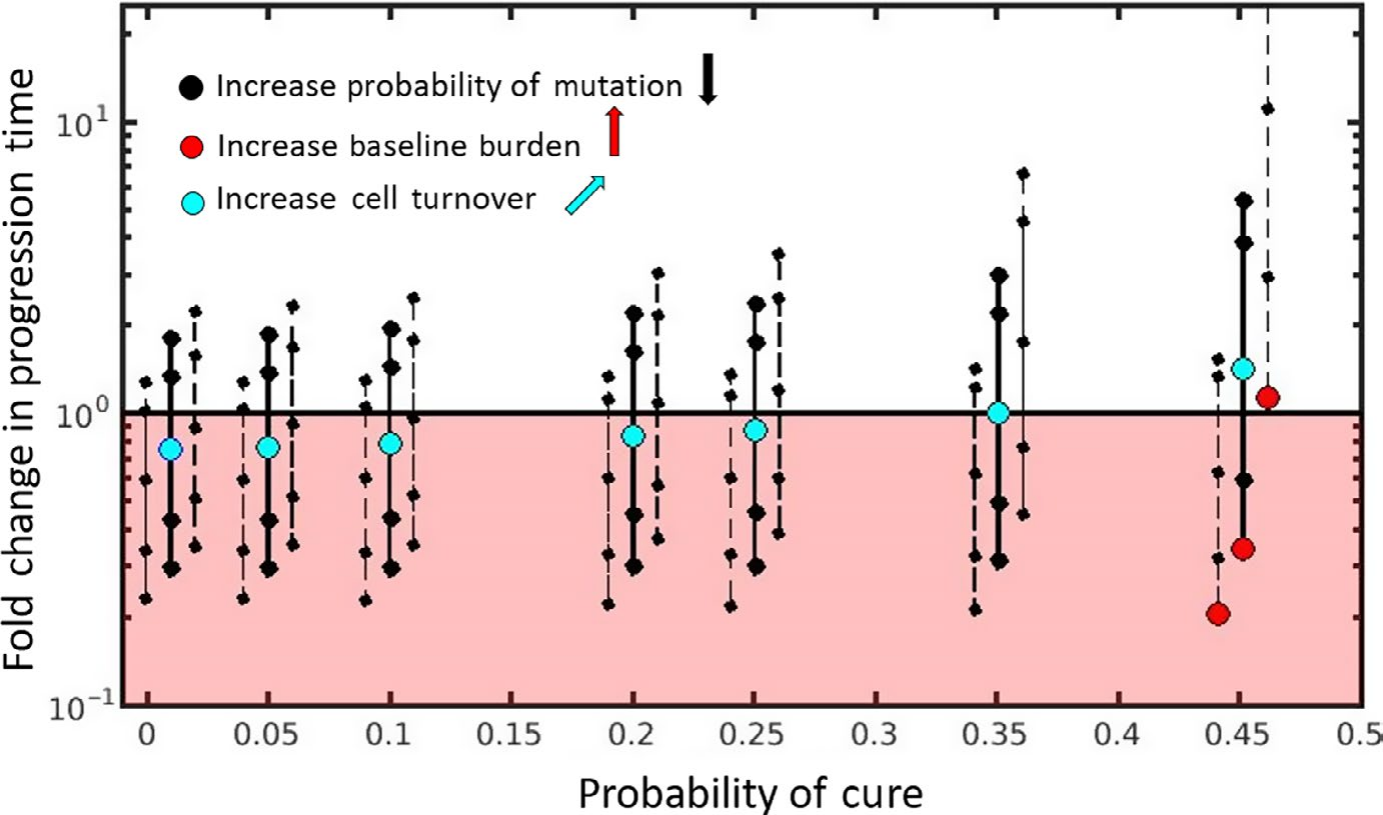
Position on cure–progression plane depends on ecology and evolution of cancer cell dynamics. Increasing the probability of mutation decreases the patient's vertical position but does not affect their horizontal position (solid black lines). A patient's vertical position is also impacted by the baseline burden (each triplet of black lines shows how a patient's vertical position changes as the baseline burden changes from low (left dashed line) to intermediate (middle solid line) to high (right dashed line). Changing the baseline burden has negligible effect on the probability of cure (the lines in each triplet are separated horizontally simply for clarity). The probability of cure for each triplet is indicated by the middle solid line. Red circles show how increasing the baseline burden from low (left‐most red circle) to high (right‐most red circle) can move a patient from the lower half to the upper half of the plane. Light blue circles show how increasing intrinsic cell turnover from low (left‐most blue circle) to high (right‐most blue circle) increases both the probability of cure and the fold change in progression time. Box 3 provides the analytic expressions for the probability of cure and the times to progression for both containment and aggressive treatment. See Box 4 for simulation details

Two main features of cell dynamics that determine a patient's position on the cure–progression plane are: (a) the balance between mutation and competition and (b) the intrinsic rate of cell turnover. Figure 3 shows how the probability of cure and the fold change in progression time depends on these details (see Box 3 for mathematical details and Box 4 for simulation details). We now take each of these features in turn.

BOX 3 **Formulas for cure and progression time**

Since aggressive treatment immediately removes all drug‐sensitive cells, the total tumor is instantly reduced to just the drug‐resistant population. It then remains to track this drug‐resistant population over time and determine: (a) how likely it is to shrink to zero (i.e., determine the probability of cure) and (b) how long it is expected to take for the tumor to grow to the baseline burden *B*_*base*_ if it does not shrink to zero (i.e., determine the expected time to progression if cure is not achieved). For aggressive treatment, we will model the dynamics of the resistant population as a Markov chain with two absorbing boundaries (one representing cure when *R* = 0 and one representing progression when *R* = *B*_*base*_). This allows us to derive analytic expressions for the probability of cure and also the expected time to progression (Ravindran et al., 1987). If *R* is the number of resistant cells and *B_A_*(*R*) and *D_A_*(*R*) are the birth and death rates after aggressive treatment, then the probability of cure when there are initially *R*_0_ resistant cells is

$$P_{R_{0}}\left(\text{cure}\right)=\frac{\sum_{q=R_{0}+1}^{B_{\mathrm{base}}-1}\prod_{z=q}^{B_{\mathrm{base}}-1}\frac{B_{A}\left(z\right)}{D_{A}\left(z\right)}+1}{\sum_{q=1}^{B_{\mathrm{base}}-1}\prod_{z=q}^{B_{\mathrm{base}}-1}\frac{B_{A}\left(z\right)}{D_{A}\left(z\right)}+1}.$$

If, on the other hand, aggressive treatment fails to cure, then the expected time to progression is

$$E_{R_{0}}^{A}\left(t_{f}\right)=\sum_{m=R_{0}}^{B_{\mathrm{base}}-1}\left[\sum_{n=1}^{m}\frac{1}{B_{A}\left(n\right)}\left(\prod_{z=n+1}^{m}\frac{D_{A}\left(z\right)}{B_{A}\left(z\right)}\right)\frac{{\left(1-P_{n}\left(\mathrm{cure}\right)\right)}^{2}}{\left(1-P_{m}\left(\mathrm{cure}\right)\right)\left(1-P_{m+1}\left(\mathrm{cure}\right)\right)}\right].$$

In the case of containment, we are still interested in determining how long it takes for the resistant population to reach the baseline burden, but we also assume that the sensitive population has the ability to directly contribute to the resistant population (e.g., through mutation). This means that the *R* = 0 boundary is no longer absorbing, and we have a birth–death process with a single absorbing boundary at *R* = *B*_*base*_. In this case, the expected time to progression when there are initially *R*_0_ resistant cells is

$$E_{R_{0}}^{C}\left(t_{f}\right)=\sum_{m=R_{0}}^{B_{\mathrm{base}}-1}\left[\sum_{n=1}^{m}\frac{1}{B_{C}\left(n\right)}\left(\prod_{z=n+1}^{m}\frac{D_{C}\left(z\right)}{B_{C}\left(z\right)}\right)\right]+\sum_{m=R_{0}}^{B_{\mathrm{base}}-1}\frac{1}{B_{C}\left(0\right)}\left(\prod_{z=1}^{m}\frac{D_{C}\left(z\right)}{B_{C}\left(z\right)}\right),$$

where *B_C_*(*R*) and *D_C_*(*R*) are the birth and death rates under containment.

BOX 4 **Simulation details**

**FIGURE 4:**
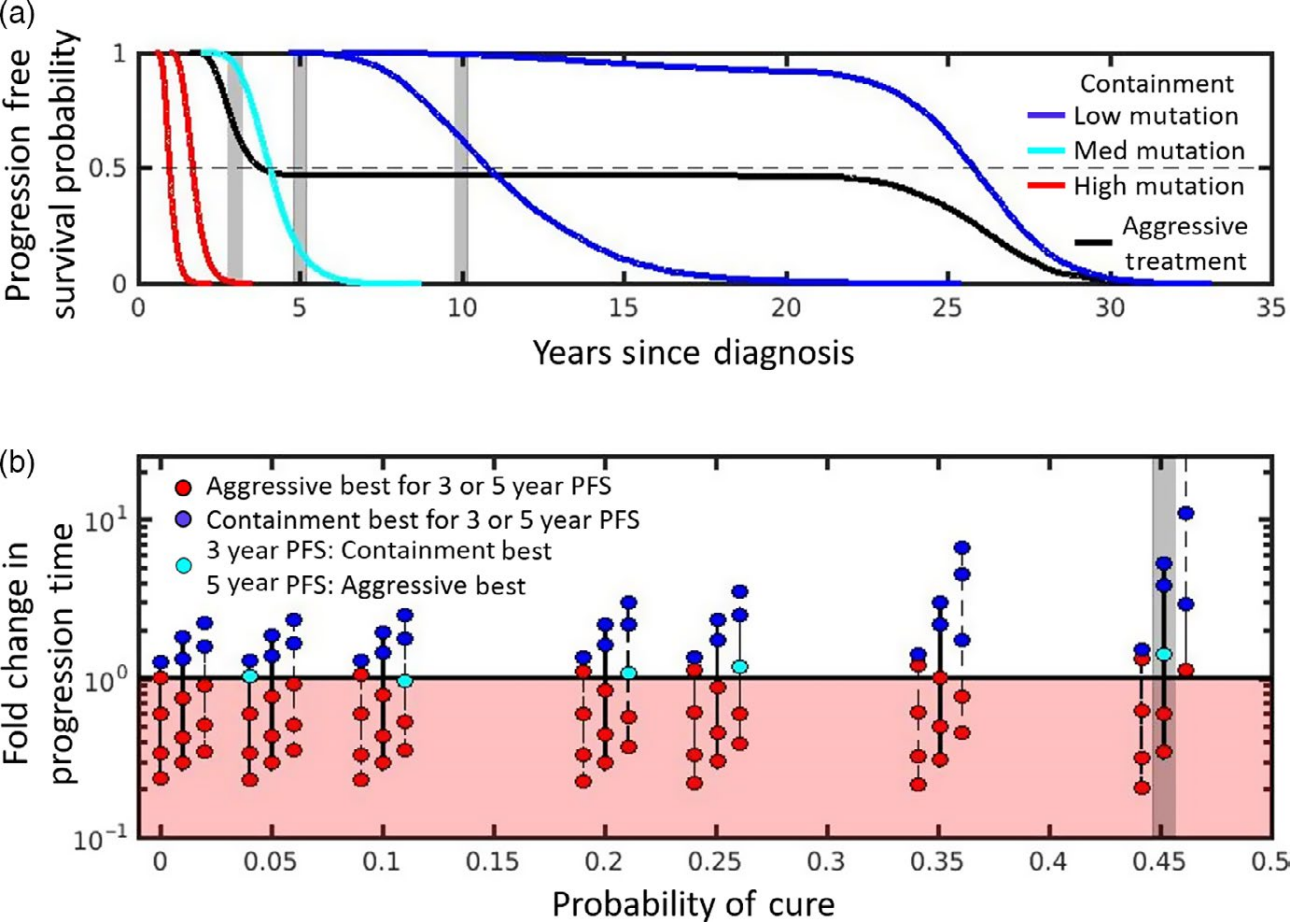
Probability of progression‐free survival (PFS) depends on evolutionary and ecological factors. Different comparison methods lead to different conclusions. Panel a: The PFS curves for containment (colored curves) and aggressive treatment (black curve) can have very different shapes. Median PFS times are determined by where curves cross the black horizontal dashed line. PFS at 3, 5, and 10 years is determined by where curves intersect the shaded areas. For high mutation probabilities (red curves), containment leads to worse PFS (by all four comparison methods). For low mutation probabilities (dark blue curves), containment leads to better PFS (by all four comparison methods). For an intermediate probability of mutation (light blue curve), whether containment or aggressive treatment is best depends on the comparison method. Curves correspond to the positions indicated by shaded area in Panel b. Panel b: Same as Figure 3 except colors now indicate how aggressive treatment and containment compare for different measures of PFS. Whether containment or aggressive treatment is best depends on whether PFS survival is measured at 3 or 5 years. In some cases, aggressive treatment is always preferred (red circles), never preferred (dark blue circles), or preferred only if PFS at 5 years is used (light blue circles). Parameter values used are identical to those used in Figure 3. Shaded area indicates the scenarios used to generate the curves in Panel a. See Box 4 for simulation details

How different aspects of tumor cell ecology and evolution impact the probability of cure and the fold change in progression time are fully specified by the analytic expressions in Box 3. Simulations are used simply to provide a visual representation of these relationships, with a focus on relative effects. Simulations are not meant to describe realistic tumor dynamics. In particular, small tumor sizes are used to allow a wide exploration of parameter values within a reasonable time frame. To compensate for the small tumor size, we use larger mutation probabilities than would be expected. However, to ensure all possible dynamics are captured, we also consider the case where there is no possibility of mutation (*ɛ* = 0). The mutation rates used in Figure 3 are 0, 0.01, 0.1, 0.45, and 1. The competition coefficient *δ* determines how large a tumor can be before cell replication is no larger possible (see Box 1 for details). For both aggressive treatment and containment, cell replication will decrease to zero when the tumor burden reaches $\frac{1}{\delta }$ . For each triplet in Figure 3, the baseline burdens are (from left to right within each triplet) $\frac{0.25}{\delta }$ , $\frac{0.5}{\delta }$ , and $\frac{0.6}{\delta }$ . Each triplet represents a different intrinsic cell turnover. From the leftmost triplet to the rightmost triplet, intrinsic cell turnover is 0.01, 0.05, 0.1, 0.2, 0.25, 0.35, and 0.45. For all simulations, the competition coefficient was set to $\delta =\frac{1}{500}$ . Each curve in Figure 4a is generated using 5,000 patients, which are diagnosed at the age of 50 and whose life spans are described by a normal distribution with a mean of 76 years and a standard deviation of 2 years. Using a large number of patients (5,000) helps to ensure that the entire distribution of possible outcomes is represented. Data sharing is not applicable to this article as no new data were created or analyzed in this study.

### 4.1 Placement on cure–progression plane: role of mutation and competition

The balance between mutation and competition predominantly impacts a patient's vertical position on the cure–progression plane. Simply by adjusting the amount of mutation, it is possible to change whether or not containment delays progression (3). Each solid black vertical line shows how decreasing the probability of mutation (while keeping all other cell properties fixed) increases the vertical position of the patient. Changing the probability of mutation (*ɛ*) does not change the patient's horizontal position because we are assuming that aggressive treatment immediately removes all sensitive cells. That means that the mutation rate does not change the probability of cure.

The balance between mutational input and competitive suppression also depends on the baseline burden. If the baseline burden is small enough, then the patient will be in the lower half of the cure–progression plane and aggressive treatment is the best option. In these patients, the tumor burden is never large enough to generate enough competition to make containment a useful strategy. By increasing the baseline burden, a patient can move from the lower half to the upper half of the plane (Figure 3, red circles moving from left to right). Each triplet of vertical lines shows how changing the baseline burden (while keeping all other properties fixed) changes the patient's position on the plane. Changing the baseline has negligible effect on the probability of cure (the dashed lines in each triplet have the same probability of cure as the solid line, they are displaced horizontally from the solid line only for clarity).

### 4.2 Placement on cure–progression plane: role of intrinsic cell turnover

A patient's position on the plane also depends on the probability of cell death *µ* and the intrinsic replication rate *r*, but it only depends on the ratio of these quantities (i.e., $\frac{\mu }{r}$ ). This ratio, which we call intrinsic cell turnover, impacts both the probability of cure and the fold change in progression and has been shown to be important for other aspects of cancer dynamics (Bozic, Gerold, & Nowak, 2016; Waclaw et al., 2015). As cell turnover increases, cell deaths become increasingly more frequent than cell births and this increases the probability of cure (probability that the resistant population dies out by chance), so that patients with higher cell turnover are further to the right on the plane. Note that this ratio $\frac{\mu }{r}$ is also the probability of cure when there is a single resistant cell (*R*_0_ = 1) and no competition (*δ* = 0) (Athreya & Ney, 1972).

The effect that intrinsic cell turnover has on a patient's vertical position is slightly more complicated. Recall that during containment, the resistant population can spend periods of time when its expansion is enhanced (when *R* < *R*_*balance*_) and periods of time when its expansion is slowed (when *R* > *R*_*balance*_) as compared to aggressive treatment. (See section: Primer on containment, for more details.) Increasing intrinsic cell turnover increases the proportion of time spent above the balance threshold where the effect of competition exceeds the effect of mutational input (see Box 5 for a more detailed discussion).

BOX 5 **How intrinsic cell turnover impacts fold change in progression time**

Total cell turnover and intrinsic cell turnover are different. Total cell turnover describes how likely cell death is compared to cell replication and depends on the treatment strategy, the size of the resistant population, and the intrinsic cell turnover $\frac{\mu }{r}$ . Under containment, total cell turnover is

$${\left(\text{Total}\;\text{Cell}\;\text{Turnover}\right)}_{C}=\frac{\mu R}{B_{C}\left(R\right)}=\frac{\mu }{r}\frac{R}{\left(1-\delta B_{\mathrm{base}}\right)R+\varepsilon \left(1-\delta B_{\mathrm{base}}\right)\left(B_{\mathrm{base}}-R\right)}.$$

Increasing either the intrinsic cell turnover or the resistant population will increase the total cell turnover. This means that increasing intrinsic cell turnover disproportionately impacts the total cell turnover at higher *R*. This translates to increasing the expected proportion of time spent at higher *R*. Provided the baseline burden is large enough (*B*_*base*_ > *R*_*balance*_), this increases the expected proportion of time spent in the area where containment slows the expansion of the resistant population (i.e., whenever *R* > *R*_*balance*_).

An analogous argument applies for aggressive treatment. Total cell turnover for aggressive treatment is

$${\left(\text{Total}\;\text{Cell}\;\text{Turnover}\right)}_{A}=\frac{\mu R}{B_{A}\left(R\right)}=\frac{\mu }{r}\frac{1}{\left(1-\delta R\right)}.$$

This means that whenever there is a chance of containment delaying progression (*B*_*base*_ > *R*_*balance*_), then increasing intrinsic cell turnover increases this chance. Using the above argument, we would expect that whenever the baseline burden is so low that there is no chance of containment delaying progression (*B*_*base*_ < *B*_*balance*_), increasing intrinsic cell turnover would actually decrease the fold change in progression time (because the expansion of the resistant population is always enhanced by containment). There is, however, an additional effect, which allows for the possibility of increasing the fold change in progression time. During treatment, the size of the resistant population will change. If the size decreases to zero, then under aggressive treatment, cure is achieved and the path of the resistant population is terminated. Under containment, however, a sensitive population will remain, and tumor dynamics will continue until a resistant cell is introduced through mutation. This means, all else being equal, there will be a subset of paths under containment that take longer to achieve progression. Since increasing intrinsic cell turnover increases the probability of cure, increasing intrinsic cell turnover will accentuate the difference between aggressive treatment and containment. This means that even if the baseline burden is below the balance threshold, it is possible to increase the fold change in progression time—although it will always stay below 1. (Increasing intrinsic cell turnover can decrease the benefit of aggressive treatment but aggressive treatment will always be better.)

Therefore, if there is any chance of containment delaying progression (i.e., *B*_*base*_ > *R*_*balance*_), increasing cell turnover will increase the fold change in progression time. This means that if containment already delays progression, increasing cell turnover will delay progression even more (patients in the upper half of the plane will move further up the plane). Additionally, even if containment causes early progression, increasing cell turnover could change this. Patients could transition from the lower half to the upper half of the plane (Figure 3, light blue circle moves upwards and to the right). See Box 5 for the case when containment has no chance of delaying progression (*B*_*base*_ < *R*_*balance*_). In conclusion, if there is any chance of containment delaying progression (the baseline burden exceeds the balance threshold), then increasing cell turnover both increases the probability of cure and allows containment to successfully delay progression for longer and for a wider range of mutation rates. This means that as cure becomes more likely, the cost of failed cure increases—making the decision between containment and aggressive treatment even more difficult.

## 5 THE DECISION BOUNDARY

Knowing when to “attempt cure” as opposed to “delay progression” requires partitioning the upper half of the cure–progression plane (Figure 2a) into areas where containment should be used and areas where aggressive treatment should be used. Or, in other words, to fully specify treatment decisions, it is necessary to define a decision boundary. One way to do this is to directly compare the benefits of cure and delayed progression by measuring how they affect the life span of the patient. Modifications of this metric might include some quantitation of the quality of life span, but for simplicity, we focus on life span per se.

If *E^C^*(LS) and *E^A^*(LS) denote the expected life span of a patient under containment and aggressive treatment, respectively, then containment would be chosen whenever

(2) $$E^{C}\left(\text{LS}\right)>E^{A}\left(\text{LS}\right).$$

For example, suppose that (a) progression is closely followed by patient death (a modified approach could be used if second‐line therapies were possible) and (b) cure returns a patient to their usual (cancer‐free) expected life span. In this case, the expected life span under aggressive treatment is

(3) $$\underset{\begin{array}{c} \text{expected}\;\text{life span}\\ \text{under}\;\text{aggressive}\\ \text{treatment} \end{array}}{\underbrace{E^{A}\left(\text{LS}\right)}}=\underset{\begin{array}{c} \text{probability}\;\\ \text{of}\;\text{cure} \end{array}}{\underbrace{P_{R_{0}}\left(\text{cure}\right)}}\underset{\begin{array}{c} \text{expected}\\ \text{natural}\;\text{life span} \end{array}}{\underbrace{E\left(\text{NLS}\right)}}+\underset{\begin{array}{c} \text{probability}\;\text{that}\\ \text{aggressive}\;\text{treatment}\\ \text{does}\;\text{not}\;\text{lead}\;\text{to}\;\text{cure} \end{array}}{\underbrace{\left(1-P_{R_{0}}\left(\text{cure}\right)\right)}}\underset{\begin{array}{c} \text{expected}\;\text{time}\;\text{to}\\ \text{progression}\;\text{when}\\ \text{aggressive}\;\text{treatment}\\ \text{does}\;\text{not}\;\text{lead}\;\text{to}\;\text{cure} \end{array}}{\underbrace{E_{R_{0}}^{A}\left(t_{f}\right)}}$$

and under containment is

(4) $$\underset{\begin{array}{c} \text{expected}\;\text{life span}\;\\ \text{under}\;\text{containment} \end{array}}{\underbrace{E^{C}\left(\text{LS}\right)}}=\underset{\begin{array}{c} \text{expected}\;\;\text{time}\;\text{to}\;\\ \text{progression}\;\text{under}\;\\ \text{containment} \end{array}}{\underbrace{E_{R_{0}}^{C}\left(t_{f}\right)}}.$$

Substituting Equations 3 and 4 into Equation 2, we see that aggressive treatment should be used whenever

$$\left(\begin{array}{c} \text{Fold}\;\text{Change}\;\text{In}\\ \text{Progression}\;\;\text{Time} \end{array}\right)=\frac{E_{R_{0}}^{C}\left(t_{f}\right)}{E_{R_{0}}^{A}\left(t_{f}\right)}<\left(\frac{E\left(\text{NLS}\right)}{E_{R_{0}}^{A}\left(t_{f}\right)}-1\right)P_{R_{0}}\left(\text{cure}\right)+1.$$

This criterion defines a linearly increasing decision boundary as shown in Figure 2b.

The slope of this line is ( $\frac{E\left(\text{NLS}\right)}{E_{R_{0}}^{A}\left(t_{f}\right)}-1$ ) and so depends on both the natural life span of the patient (*E*(NLS)) and the expected time to progression when aggressive treatment fails to cure the patient ( $E_{R_{0}}^{A}\left(t_{f}\right)$ ). For example, all else being equal, the decision boundary for a cancer that is diagnosed in childhood increases much more rapidly than one diagnosed in an elderly patient. Thus, aggressive treatment should be used in a childhood disease even if the probability of cure is fairly low because of the great benefit received in the unlikely event that the child is cured. On the other hand, the probability of cure would have to be quite high before aggressive treatment was warranted in an older patient—because the gains associated with cure are not that much better than simply delaying progression.

This approach provides a straightforward way to compare the different outcomes of treatment, but the focus on expected (mean) life expectancy has some disadvantages. For example, if the distribution of possible outcomes is heavily skewed, the expected remaining life span may be an inadequate summary of this distribution. Importantly, it is possible for an aggressive treatment with a high probability of cure but rapid progression if it fails to result in the same expected life span as a containment strategy that consistently results in modest delays in progression. Although these strategies would be considered equivalent with this method, patients may value these outcomes very differently. As another example, in the diagnosis of childhood cancer mentioned above, patients may prefer the certainty of modest gains over the unlikely possibility of very large gains.

Here, we have described a possible method for defining a decision boundary and discussed some of its shortcomings. There are many ways to make this decision and no single best choice for all scenarios. Ultimately, the treatment decision will depend on two factors: (a) the patient's location on the cure–progression plane and (b) how “cure” and “delaying progression” are prioritized.

## 6 COMPARING STRATEGIES IN CLINICAL TRIALS

How “cure” and “delaying progression” are prioritized also has important implications not only for the patient, as above, but also for the comparison of containment and aggressive treatment in clinical trials. In order to be approved, containment strategies must show improved (or at least noninferior) outcomes to standard aggressive treatments and it has become increasingly common to use *progression‐free survival* (PFS) to do this (Korn & Crowley, 2013; Seymour et al., 2010; Villaruz & Socinski, 2013). PFS is the time to progression or death from any cause (Green, Benedetti, & Crowley, 1997). Because PFS includes both progression and death, the distribution of event times in a trial can be quite skewed. For example, with aggressive treatment the time to progression for patients who are not cured can be much shorter than the time to death for patients who are successfully cured. The presence of these two sets of patients (cured vs. those who progress) can make comparison of PFS for containment and aggressive treatment difficult. Different comparison methods prioritize cure and delayed progression differently.

Progression‐free survival data are often summarized using the Kaplan–Meier curves (Rich et al., 2010). There are a number of summary statistics commonly used to compare these curves. Examples include median PFS, PFS at certain years (i.e., 3, 5, or 10 years), and statistical tests that compare the entire curve like the log‐rank test. Sometimes, the Kaplan–Meier curves will cross and this can make it difficult to compare them in a meaningful way (Li, Han, Hou, Chen, & Chen, 2015; Logan, Klein, & Zhang, 2008). Our conceptual framework suggests that crossing curves are especially likely to occur when comparing containment and aggressive treatments. This is best illustrated by examining PFS curves for containment and aggressive treatment under the assumption that tumor dynamics are described by the model in Box 1.

Figure 4a shows PFS curves for containment for a variety of different mutation rates (colored curves) and for aggressive treatment (black curve). The red containment curves correspond to higher probabilities of mutation, which cause rapid progression during containment. These curves lie below the black curve, and for these high mutation probabilities, aggressive treatment leads to better progression‐free survival. In this case, the choice is obvious, regardless of how cure and delayed progression are prioritized. Aggressive treatment is the better option.

The blue (light and dark) containment curves correspond to lower mutation probabilities, and they initially all show better PFS than aggressive treatment. The right‐most dark blue curve corresponds to containment under the assumption that there is no mutation. This curve never crosses the black curve and has a much higher median PFS than aggressive treatment (as indicated by where the curves cross the dashed line). It also has better PFS at 3, 5, and 10 years (compare intersection of right‐most blue curve and black curve with gray shaded areas). On the other hand, the left‐most dark blue curve initially begins above the aggressive treatment curve but eventually crosses. However, the crossing occurs sufficiently late that containment is still preferred by all 4 measures (median PFS or PFS at 3, 5, and 10 years). The picture is not as clear if the mutation rate is slightly higher. For example, although the light blue curve has approximately the same median PFS as aggressive treatment, containment in this case improves PFS at 3 years but decreases PFS at 5 and 10 years. Different comparison methods lead to different conclusions. For this example, with this mutation rate, using PFS at 3 years to compare strategies prioritizes delaying progression. Comparing the curves at 5 years priorities “long‐term” survival (or cure). But this could change for different mutation rates and depends a lot on how much containment delays progression. For example, if PFS at 20 years was used to compare treatment strategies, then for the mutation rate corresponding to the left‐most dark blue curve, aggressive treatment would be better.

Figure 4b shows how treatment decisions based on PFS at 3 and 5 years depends on the probability of mutation, baseline burden, and intrinsic cell turnover. In some situations, containment is the preferred strategy regardless of whether PFS is assessed at 3 or 5 years (blue circles), in other situations, aggressive treatment is always preferred (red circles). There are also cases where the decision depends on how PFS is assessed (light blue circles). In these cases, containment is preferred when the earlier time is used (PFS at 3 years) and aggressive treatment is preferred if the later time (PFS at 5 years) is used.

Figure 4b emphasizes that there are distributions of possible progression times for each treatment approach and hence different ways to define the fold change in progression time. As mentioned previously, we have chosen to use the expected times to progression (the means of these distributions) to define the fold change in progression time, and this definition separates the cure–progression plane into two halves: the lower half (Figure 4b, shaded red area) where aggressive treatment is always best, and the upper half (Figure 4b unshaded area) where containment delays the expected time to progression. Figure 4b shows that if we had defined fold change in progression time using the probability that progression time exceeds either (a) 3 years or (b) 5 years, this would have changed how patients are divided between the upper and lower halves of the cure–progression plane.

In summary, this analysis shows that when comparing standard practice (aggressive treatment) with resistance management strategies in clinical trials, it is important to ensure that the method used to compare treatment strategies is consistent with how “cure” and “delayed progression” are prioritized.

## 7 DISCUSSION

There are many uncertainties in cancer treatment and no easy decisions. Every patient's cancer represents a unique ecology that will likely evolve and respond to treatments in different ways. Despite these difficulties, the intent of treatment used to be clear (Balis, 1998; Barlogie et al., 2008; Frei, 1985; Lonial & Anderson, 2014). The best treatment was the one that maximally reduced the tumor burden. In the best case, this would cure the patient, and in the worst case, it would at least slow the progression of the cancer. Cure was the aim, and delayed progression was the outcome when treatment failed.

Focusing on managing resistance, however, challenges this paradigm. In particular, strategies that leverage competition are designed to slow progression and work by deliberately maintaining a tumor burden (so that cure is normally not possible) (Gatenby, 2009; Hansen et al., 2017). This forces a decision between “treating to cure” and “treating to slow progression.” It is not obvious how to make this decision. Here, we focused on possible treatment responses of a single patient whose cancer dynamics are understood. By ignoring patient‐to‐patient variability and assuming we perfectly understand tumor dynamics within a patient, we are able to focus on the complexities of making treatment decisions—complexities that are inherent in the decision‐making process and not related to additional uncertainties and heterogeneities.

The decision between attempting cure and delaying progression depends on how these outcomes are prioritized. It also depends on the probability of cure and how much containment can delay treatment (a patient's position on the cure–progression plane; Figure 2b). The probability of mutation, the baseline burden, and the intrinsic cell turnover all impact a patient's position on the cure–progression plane. Another factor that is important is the number of resistant cells when the treatment decision is being made (*R*_0_). Increasing *R*_0_ will decrease the probability of cure (shifts a patient's position to the left). Additionally, the larger the *R*_0_ is, the more likely that containment delays progression (although the amount of delay is not proportional to *R*_0_). This means that containment is more likely to be the better option for tumors with a larger proportion of resistant cells. Indeed, a high *R*_0_, high baseline burden, and low mutation rate make it more likely that containment is the preferred option. On the other hand, a low *R*_0_, low baseline burden, and high mutation rate make it more likely that aggressive treatment is the better option. Choosing between aggressive treatment and containment will be most difficult when there is a high probability of cure and containment significantly delays progression (i.e., the patient lies in the upper right of the cure–progression plane). This occurs when the intrinsic cell turnover and baseline burden are high and the initial number of resistant cells and probability of mutation are low.

Of the factors impacting placement on the cure–progression plane, baseline burden is particularly interesting because it can be easily manipulated by treatment itself (and perhaps other options like surgery). Earlier, we recognized that to immediately alleviate patient suffering, the baseline tumor burden may need to be lowered to an acceptable level before considering containment. It is also possible that a patient's baseline burden could be increased before making the final treatment decision. Increasing the baseline burden can significantly increase the benefit of containment (increase a patient's vertical position on the cure–progression plane) while having negligible impact on the probability of cure. This will only be the case if the baseline burden is sufficiently large. When it is known that available treatment options cannot effect a cure, it may be advantageous to deliberately increase a patient's baseline burden and maximize the chances that containment will delay progression. This may involve withholding treatment until the baseline is sufficiently large to make containment a viable option. Indeed, the baseline burden has to be sufficiently large for this strategy to work (Hansen et al., 2017).

Our analysis used idealized versions of containment and aggressive treatment. Containment maintains the tumor burden at the baseline burden, and aggressive treatment immediately removes all sensitive cells. In practice, it will be difficult to maintain a constant tumor burden during containment. An example of a practical approximation to containment is the “adaptive therapy” strategy currently being tested in prostate cancer (Zhang et al., 2017). If maximizing competition is the aim, there may be better approximations. Another alternative is to base treatment decisions on symptoms instead of tumor burden. Here, the idea would be to treat just enough to relieve symptoms and use this as a proxy for the maximum acceptable baseline. Implementing aggressive treatment involves different difficulties. In many cases, treating aggressively will in reality remove most—but not all—sensitive cells (Fulciniti, Munshi, & Martinez‐Lopez, 2015). This means that real‐life aggressive treatment can be similar to containment implemented at a very low baseline burden. In this case, the main effect of the residual sensitives is to increase the number of resistant cells through mutation. When this occurs, “real‐life containment” should delay progression relative to “real‐life aggressive treatment,” but “real‐life aggressive treatment” could still lead to cure. So even in realistic scenarios, a choice must often be made between “attempting cure” and “delaying progression.”

The word “cure” is a fraught term in oncology. The nature of cancer is that we never really know whether it has gone away. A patient may be cured or may simply be a long‐term responder who will eventually relapse. After aggressive treatment, the tumor burden may be below detection level. An undetectable tumor burden may consist of only resistant cells, only sensitive cells, or a mixture of both. The composition of the residual tumor burden will impact the success of long‐term maintenance therapies.

In reality, treatment will reduce the number of sensitive cells indirectly by either lowering the rate of cell replication *r* or increasing the rate of cell death *µ*. This means that in addition to changing the number of sensitive cells, real‐life drug treatment will probably also increase the intrinsic cell turnover $\frac{\mu }{r}$ . Drug treatment itself can shift a patient's position on the plane (upwards and to the right). This increases the chances of being in a situation where the choice between aggressive treatment and containment is difficult.

Our analysis does not consider changes in the immune response during treatment. Changes in immunity can change a patient's position on the cure–progression plane. For example, if the immune response improves over time the tumor burden could be reduced below the original baseline burden (even in the absence of drugs). Additionally, if increased immune response increases the probability of cell death, then the intrinsic cell turnover will increase. In this case, a combined treatment strategy that uses containment to delay progression until the immune response is sufficiently strong and then implements aggressive treatment may be best. Another time when a combined treatment strategy (containment followed by aggressive treatment) may be best is when resistance carries certain types of fitness costs. A combined treatment strategy has the benefit of leveraging competition to delay progression until a patient's position on the cure–progression plane shifts to a location where “attempting cure” is the better option. A detailed consideration of changing immune response and fitness costs is beyond the scope of this analysis, but a preliminary analysis of these issues can be found in Hansen et al. (2017) and its supplemental information. We also highlight some of the key considerations regarding fitness costs in Box 6.

BOX 6 **Fitness costs associated with resistance**

As evidenced by our analysis here, fitness costs are not necessary for containment to be beneficial. Despite this, fitness costs—and how they manifest—will impact a patient's position of the cure–progression plane. If resistance carries an intrinsic fitness cost (reduced ability to replicate in the absence of competition, i.e., lower *r*), this will increase intrinsic cell turnover and will also increase the balance threshold *R*_*balance*_. On the other hand, if resistant cells have a reduced ability to compete, this will lower the balance threshold and increase the benefit of containment (Hansen et al., 2017).

When interpreting clinical trial results, heterogeneous patient populations can make understanding the outcomes of different strategies difficult. In our discussion of PFS curves, we greatly simplified these complexities by assuming that all patients were identical. This meant that any differences in response to treatment were due to stochastic effects of cell dynamics (random chance) and not intrinsic differences between patients. In particular, we assumed that all patients began with the same number of resistant cells. In practice, this will not be the case and the comparison and interpretation of PFS curves will be more difficult. This is especially true when comparing containment and aggressive treatment strategies because the choice between these is so dependent on a patient's location in the cure–progression plane. This means that in trials comparing containment and aggressive treatment, extra care should be made to account for differences in patients' initial resistant burdens. This can be difficult to do, since it is often not possible to directly measure resistant burden, but considering a patient's treatment history or possibly the rate of tumor response at the beginning of therapy may be indirect ways to estimate this burden. This also suggests that cancer–drug pairs with known resistance markers may be good candidates for initial trials of containment strategies. With good markers of resistance, it may be possible to estimate the resistant burden.

By not including patient‐to‐patient heterogeneities, our analysis emphasizes another important point; differences in treatment response are not necessarily linked to differences in patients. Due to random fluctuations in tumor cell dynamics, there is a distribution of possible responses that a patient may have. This means that even though identifying subpopulations of patients with different features will help with treatment decisions (and appropriate allocation to trial arms), the difficulty of choosing between “attempting cure” and “delaying progression” will remain.

Fundamental to choosing between attempting cure and delaying progression is understanding the value of each of these outcomes. It is difficult to determine these values a priori. One approach we explored was to use the expected remaining life span. In our exploration, we greatly simplified things by assuming that tumor progression is closely followed by death. Generally, the link between progression and time to death (i.e., overall survival) is not well understood. Sometimes, increasing time to progression increases overall survival; other times, this trend is reversed (Beauchemin, Johnston, Lapierre, Aissa, & Lachaine, 2015; Foster et al., 2011; Pfeiffer, Hashim, Duran, Postma, & Heeg, 2017). In many cases, the relationship between delayed progression and overall survival is simply unknown. It is possible that the baseline used to measure progression affects this relationship, with delayed progression at higher baselines being more closely linked to improved overall survival.

Even within a single patient with well‐understood tumor dynamics, many factors influence the relative value of cure and delayed progression. Because of these difficulties, it is important to be explicit about the fact that making a treatment decision requires choosing between attempting cure and delaying progression. Not being explicit about this choice can lead to poor decisions. This is true when communicating treatment options to patients, and it is also true when setting treatment guidelines and evaluating data from clinical trials. Before the advent of evolutionary strategies aimed at resistance management, there was often no choice. The aims of cure and delayed progression coincided. Now that there is a choice, it is critical we reformulate our decision‐making processes to reflect this.

## CONFLICT OF INTEREST

None declared.

## Data Availability

Data sharing is not applicable to this article as no new data were created or analyzed in this study.
